# Incidence of fistula occurrence in patients with cervical cancer treated with bevacizumab: data from real-world clinical practice

**DOI:** 10.1007/s10147-022-02196-8

**Published:** 2022-06-27

**Authors:** Toru Sugiyama, Noriyuki Katsumata, Takafumi Toita, Masako Ura, Ayaka Shimizu, Shuichi Kamijima, Daisuke Aoki

**Affiliations:** 1 St. Mary’s Hospital, 422 Tsubukuhonmachi, Kurume, Fukuoka 830-8543 Japan; 2 Department of Medical Oncology, Nippon Medical School, Musashikosugi Hospital, 1-396 Kosugi-machi, Nakahara-ku, Kawasaki, Kanagawa 211-8533 Japan; 3 Radiation Therapy Center, Okinawa Chubu Hospital Okinawa, 281 Miyazato, Uruma, Okinawa 904-2293 Japan; 4 Oncology Lifecycle Management Department, Chugai Pharmaceutical Co., Ltd., 2-1-1 Nihonbashi-Muromachi, Chuo-ku, Tokyo, 103-8324 Japan; 5 Real World Data Science Department, Chugai Pharmaceutical Co., Ltd., 2-1-1 Nihonbashi-Muromachi, Chuo-ku, Tokyo, 103-8324 Japan; 6 Medical Science Department, Chugai Pharmaceutical Co., Ltd., 2-1-1 Nihonbashi-Muromachi, Chuo-ku, Tokyo, 103-8324 Japan; 7 Department of Obstetrics and Gynecology, Keio University School of Medicine, 35 Shinanomachi, Shinjuku-ku, Tokyo, 160-8582 Japan

**Keywords:** Uterine cervical neoplasms, Bevacizumab, Health surveys, Fistula

## Abstract

**Background:**

This study aimed to determine the incidence of pelvic fistulas in cervical cancer patients treated with bevacizumab in Japanese clinical practice.

**Methods:**

A post-marketing surveillance (PMS) study was conducted between June 2016 and February 2018 to survey physicians who treated advanced or recurrent cervical cancer patients with bevacizumab (according to the product label). The clinical/treatment status of patients with pelvic fistulas was assessed in an additional retrospective case series study.

**Results:**

142 patients were included in the PMS study (median age 51 years; 66.9% squamous cell carcinoma; 66.2% recurrent cervical cancer; 64.1% previous radiotherapy). Patients received a median of seven bevacizumab doses. Six patients, all of whom had a history of pelvic irradiation, developed seven fistulas (4.2%; 95% confidence interval, 1.56–8.96), and five patients had also undergone pelvic surgery. The case series study of the patients who developed fistulas indicated that three patients had high cumulative bladder and rectal doses of radiation, and two of them had undergone salvage re-irradiation for pelvic recurrence. The other three patients underwent both radical hysterectomy and adjuvant radiotherapy, but did not receive an excessive radiation dose to the bladder or rectum.

**Conclusions:**

This study found that the upper limit of the 95% confidence interval for pelvic fistula incidence did not exceed the incidence reported in the GOG 240 study. To ensure an adequate benefit-risk assessment of bevacizumab in cervical cancer patients, a comprehensive evaluation of prior treatment is essential and the possibility of unexpected fistulas, even after careful evaluation, should be considered.

**Supplementary Information:**

The online version contains supplementary material available at 10.1007/s10147-022-02196-8.

## Introduction

Globally, cervical cancer is the fourth most common cancer and the fourth most common cause of cancer-related death in women [1, 2]. Age-standardized 2018 incidence rate estimates are 13.3 per 100,000 women per year worldwide [2]. In Japan, approximately 12,800 women are diagnosed with cervical cancer and approximately 4200 die from it each year [2].

Early-stage cervical cancer can be treated with surgery, radiation, or concurrent chemoradiotherapy (CCRT), while locally advanced cervical cancer is treated with CCRT [3]. Patients with recurrent, persistent or metastatic disease have limited treatment options [3] and a poor prognosis [4–6]. The phase 3, randomized GOG 240 study (*n* = 452) investigated the addition of bevacizumab to combination chemotherapy in this patient group, and reported improved survival and response versus chemotherapy alone [7]. A subsequent phase 2 study in Japanese patients with stage IVB persistent or recurrent cervical cancer (study JO29569, *n* = 7) found that cisplatin, paclitaxel and bevacizumab combination therapy was tolerable and had encouraging activity [8]; the results of this study and the GOG 240 study led to the approval of bevacizumab for the treatment of advanced or recurrent cervical cancer in Japan in 2016 [9]. New treatments, such as immune checkpoint inhibitors, are in late-stage development for cervical cancer in studies such as KEYNOTE-826 (NCT03635567) [10] and BEATcc (NCT03556839) [11] and it is likely that their use will improve outcomes further. These novel agents are likely to be used in combination with bevacizumab, so it is important to gather ongoing safety data on bevacizumab, which will remain a key drug for the treatment of cervical cancer.

With regard to safety, the GOG 240 study conducted mostly in the United States (US) identified an increased rate of fistula occurrence in the bevacizumab plus chemotherapy group compared with the chemotherapy alone group, both in the original publication and the final analysis (overall: 15% vs 1%; grade 3: 6% vs < 1%) [7, 12]. Although no fistulas were reported in the JO29569 study [8], the study included only seven patients and incidence of fistula development in Japanese women with cervical cancer remains unknown. This paper reports the results of a post-marketing surveillance (PMS) study and a case series study that aimed to determine the incidence of adverse reactions related to pelvic fistulas in bevacizumab-treated patients in clinical practice in Japan.

## Materials and methods

### Study rationale and design

At the time of bevacizumab approval for advanced or recurrent cervical cancer in Japan, the regulatory authority determined that a PMS study was necessary to confirm the safety of bevacizumab in Japanese patients with cervical cancer (UMIN registration number: UMIN000022386). This PMS study was conducted between June 2016 and February 2018 in accordance with Strengthening the Reporting of Observational Studies in Epidemiology (STROBE) Statement [13], and the Japanese regulatory requirements stipulated in the Good Post-marketing Study Practice Ministerial Ordinance in Japan (GPSP). The requirement for Institutional Review Board/Ethics Committee (IRB/IEC) approval was waived under GPSP. Although the need for informed consent to participate in this PMS study was also waived under GPSP, informed consent was obtained for all patients receiving bevacizumab according to the product label.

An additional case series study (UMIN registration number: UMIN000040043) was subsequently conducted in five patients with pelvic fistulas who consented to the collection and analysis of additional data. The aim of this study was to ascertain further details regarding fistula occurrence and treatments before bevacizumab administration to review the clinical course of patients with fistula development. This study was conducted in compliance with the Declaration of Helsinki and the Ethical Guidelines for Medical Research in Humans, and approved by the IRB/IEC of the Non-Profit Organization MINS, St. Mary’s Hospital and institutions where patients’ medical records were collected. Written informed consent or opt-out method was applied for all patients according to the Japanese Ethical Guidelines for Medical and Health Research Involving Human Subjects.

### Patients and data collection

For the initial PMS study, a survey of physicians treating cervical cancer patients with bevacizumab according to the product label (bevacizumab 15 mg/kg every 3 weeks via intravenous infusion) was conducted. The survey included questions on patient background, administration conditions, current treatment status, concomitant medications, combination chemotherapy, occurrence of pelvic fistulas, and details regarding the fistulas that occurred. Information on the adverse event (AE) “fistulas in the pelvis” was collected; other data on AEs were not collected. Reported events, including fistulas, were graded by physicians according to the National Cancer Institute Common Terminology Criteria for Adverse Events version 4.0.

For the case series study, additional data were sought from the medical records of the six patients with cervical cancer who developed a pelvic fistula with bevacizumab, and data from five patients were collected and analyzed. Relevant data were collected on the characteristics of patients, such as blood test findings, details of previous treatment including surgery, procedures, and radiotherapy (Online Resource Table S1). To supplement the radiotherapy evaluation, actuarial treatment planning data were collected, e.g. digital reconstruction images and computed tomography (CT) images with dose distribution. Data on treatment and outcomes after fistula development were also collected.

### Statistical analysis

The target population size for the initial survey was 130 patients, based on the overview of fistula incidence contained in the review report on bevacizumab prepared by the Japanese Pharmaceuticals and Medical Devices Agency [14]. Data were analyzed descriptively and presented as proportion or number of patients, means and standard deviations, Clopper–Pearson 95% confidence intervals (CIs), or median, as applicable. No statistical analysis was conducted on data from the case series study, which was purely descriptive in nature.

## Results

### Post-marketing surveillance study

**Fig. 1:**
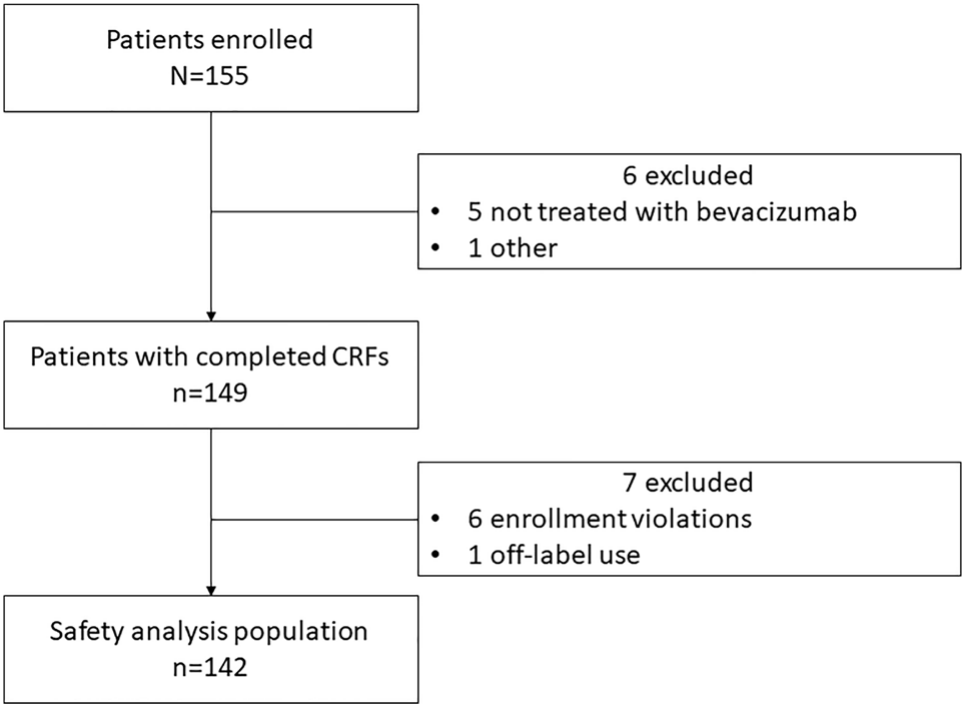
Disposition of patients included in the survey. *CRFs* case report forms

In total, 98 centers that treated 155 cases of cervical cancer were included in the PMS study; of these, data for 149 patients were collected, and 142 patients were included in the safety analysis (Fig. 1). The median age of the cohort was 51 years and 66.9% of the patients had squamous cell carcinomas. 97.2% had tumor lesions at baseline, and 64.1% had undergone previous radiotherapy (8.5% radiation monotherapy, 51.4% CCRT, and 4.2% both). Overall, 35.2% of patients had a recurrence in the radiation field at the start of bevacizumab treatment (Table 1).

**Table 1:** Characteristics of the patients included in the post-marketing surveillance study

Characteristic	Total population (*N* = 142)
Age, years	
Mean (SD)	51.1 (12.0)
Median (range)	51.0 (24–84)
BMI, *n* (%)	
< 18.5 kg/m^2^	25 (17.6)
18.5 to < 25 kg/m^2^	87 (61.3)
≥ 25 kg/m^2^	28 (19.7)
Unknown/not stated	2 (1.4)
GOG PS category, *n* (%)	
0	104 (73.2)
1	35 (24.6)
≥ 2	3 (2.1)
Tumor histology, *n* (%)	
Squamous cell carcinoma	95 (66.9)
Adenocarcinoma	35 (24.6)
Adenosquamous carcinoma	5 (3.5)
Other	7 (4.9)
Treatment line of chemotherapy, *n* (%)	
First	62 (43.7)
Second	52 (36.6)
Third	22 (15.5)
Other	6 (4.2)
Onset and recurrence, *n* (%)	
First occurrence^a^	48 (33.8)
Stage IIIB	3 (2.1)
Stage IVA	0 (0.0)
Stage IVB	41 (28.9)
Other	4 (2.8)
Recurrence	94 (66.2)
Tumor lesions at baseline, n (%)	
No	4 (2.8)
Yes	138 (97.2)
Pelvic lymph node	56 (39.4)
Extrapelvic lymph nodes	65 (45.8)
Vagina	14 (9.9)
Uterus	54 (38.0)
Lung	35 (24.6)
Liver	16 (11.3)
Rectum	3 (2.1)
Colon	1 (0.7)
Peritoneal dissemination	17 (12.0)
Bladder	2 (1.4)
Bone	16 (11.3)
Other	10 (7.0)
Previous radiotherapy, *n* (%)	
No	51 (35.9)
Yes	91 (64.1)
Recurrence in the radiation field at the start of bevacizumab therapy	
No	41 (28.9)
Yes	50 (35.2)
Type of previous radiation-related treatment, *n* (%)	
Radiation monotherapy	12 (8.5)
Brachytherapy	3 (2.1)
External beam radiotherapy	13 (9.2)
Brachytherapy and external beam radiotherapy	2 (1.4)
Concurrent chemoradiotherapy	73 (51.4)
Brachytherapy	1 (0.7)
External beam radiotherapy	35 (24.6)
Brachytherapy and external beam radiotherapy	43 (30.3)
Unknown/not stated	1 (0.7)
Radiation monotherapy plus concurrent chemoradiotherapy	6 (4.2)

*BMI* body mass index, *GOG PS* Gynecologic Oncology Group performance status, *SD* standard deviation

^a^Staged according to FIGO 2008 [40]

Patients received a median of seven bevacizumab doses over a median treatment period of 136 days. The mean total dose over the treatment period was 102.01 ± 37.6 mg/kg (Table 2). At the time of survey completion, 68 patients (47.9%) were still receiving bevacizumab and 74 patients (52.1%) had discontinued. Overall, 141 patients (99.3%) received bevacizumab in combination with other therapies, most commonly paclitaxel (received by 95.1% of patients), followed by carboplatin (63.4%), cisplatin (34.5%) and nogitecan (0.7%). Paclitaxel/carboplatin was the most common combination therapy (Table 2).

**Table 2:** Bevacizumab treatment course and chemotherapies received by the survey cohort

	*N* = 141
Number of doses of bevacizumab	
Mean (SD)	6.8 (2.5)
Median (range)	7.0 (2–12)
Median (range) bevacizumab treatment period, days	136.0 (3–234)
Mean (SD) daily dose of bevacizumab, mg/kg	14.99 (0.14)
Mean (SD) total dose of bevacizumab, mg/kg	102.01 (37.6)
Chemotherapies received, *n*^a^	
Paclitaxel/cisplatin	48
Paclitaxel/carboplatin	88
Paclitaxel/nogitecan	1
Other	12
Irinotecan monotherapy	2
Etoposide monotherapy	2
Carboplatin monotherapy	1
Denosumab monotherapy	1
Docetaxel/carboplatin	2
Docetaxel/cisplatin	1
Docetaxel monotherapy	1
Nedaplatin/irinotecan	1
Nedaplatin monotherapy	1
Paclitaxel monotherapy	2

*SD* standard deviation

^a^Patients could have received multiple types of chemotherapies

A total of seven fistulas occurred in six patients (4.2%; 95% CI 1.56–8.96), aged 35–61 years (median 54 years). Clinical characteristics of these patients are shown in Table 3. Fistulas developed in five of the 50 patients (10.0%) who had recurrence in the radiation field at the start of bevacizumab administration, and in one patient of the 41 (2.4%) without recurrence in the radiation field. The incidence of fistulas by treatment line was 3.22% (2/62 of cases), 5.76% (3/52 of cases), and 4.54% (1/22 of cases) for first-, second- and third-line treatment, respectively.

**Table 3:** Characteristics of patients with cervical cancer who developed a pelvic fistula with bevacizumab, and their chemotherapeutic treatment for cervical cancer prior to, and for, recurrence

Characteristic	Patient 1	Patient 2^a^	Patient 3	Patient 4	Patient 5	Patient 6
Age, years	41	54	61	35	53	55
BMI, kg/m^2^	24.1	13.7	21.0	18.3	24.6	19.3
GOG PS category	1	0	0	0	1	0
Tumor histology	SCC	SCC	SCC	SCC	SCC	SCC
Tumor stage^b^ at initial diagnosis	IIB	IB	IIIA	IIB	IIIB	IIB
Site of recurrence at bevacizumab initiation	Pelvic lymph node	Rectum	Vagina	Peritoneum^c^, intrapelvic (left dorsal)	Vagina	Extrapelvic lymph nodes
Site of fistula^d^	Vagina and rectum	Vagina and rectum	Vagina and ureter	Vagina and bladder	Vagina and bladder / vagina and rectum	Right ureter and right common iliac artery
Relationship between fistula site and cancer lesion site during bevacizumab^e^	Not related	Fistula at lesion site	Fistula around lesion	Not related	Fistula at lesion site/ fistula at lesion site	Not related
No. of bevacizumab cycles	4	NA	7	5	3	4
Combination therapy	Paclitaxel + carboplatin	Paclitaxel + cisplatin	Paclitaxel + carboplatin	Paclitaxel + cisplatin	Paclitaxel + carboplatin	Paclitaxel + carboplatin
Antitumor response of bevacizumab-containing chemotherapy	PR	NA	PR	SD	PR	CR

*BMI* body mass index, *CCRT* combined chemotherapy and radiotherapy, *CR* complete response, *GOG PS* Gynecologic Oncology Group performance status, *NA* not available, *PR* partial response, *RT* radiotherapy, *SD* stable disease

^a^Patient 2 column indicates the patient whose data were not further investigated in the retrospective case series study and data from PMS study are listed

^b^Staged according to FIGO 2008

^c^Patient had peritoneal dissemination

^d^Fistula could cover multiple sites

^e^Options in the questionnaire were as follows: Fistula at lesion site, fistula around lesion, fistula in pelvis at a site unrelated to lesion

Of the six patients with fistulas, five could be investigated further to collect additional detailed information. These five patients are the focus of the case series study.

### Case series study

Surgical and radiotherapy history of the six patients with fistulas are shown in Table 4, and the type and clinical course of their fistulas in Table 5. Patient 2 was not included in the case series study, but data from the PMS study are listed in these tables. Six vaginal fistulas occurred in five patients (3.5%); these were fistulas between the vagina and rectum (patients 1, 2 and 5), vagina and bladder (patients 4 and 5), and vagina and ureter (patient 3). Genitourinary fistulas occurred in three patients (vagina to bladder in patients 4 and 5, and vagina to ureter in patient 3) (Table 5). All fistula occurrences were considered serious; two were grade 2, four were grade 3 and one was grade 4, but there were no grade 5 fistulas causing death. All patients with fistulas had a history of pelvic irradiation (Tables 3 and 4).

**Table 4:** Details of the prior surgical and radiotherapy treatments undertaken in patients with cervical cancer in the patients who developed a pelvic fistula with bevacizumab

Characteristic	Patient 1	Patient 2^a^	Patient 3	Patient 4	Patient 5	Patient 6
Surgery						
Type of surgery	Pelvic lymph node dissection	Radical hysterectomy, pelvic lymph node dissection, and bilateral salpingo-oophorectomy	None	Radical hysterectomy, pelvic lymph node dissection, and bilateral salpingo-oophorectomy	Simple hysterectomy	Radical hysterectomy
Intent of surgery	Salvage	Curative/initial	–	Curative/initial	Salvage	Curative/initial
Duration of surgery	4 h 33 min	NA	–	6 h 42 min	2 h 20 min	5 h 40 min
Intraoperative blood loss, mL	314	NA	–	1774	175	2170
Other interventions	–	NA	–	Ureteral stenting	–	Ureteral stenting
Radiotherapy						
Type of radiotherapy	Definitive CCRT	Adjuvant CCRT	Definitive CCRT	Adjuvant RT	Definitive CCRT	Adjuvant CCRT
External beam radiotherapy WP/CS-WP	30 Gy/20 Gy	50 Gy/0	40 Gy/10 Gy	50 Gy/0	39.6 Gy/10.8 Gy	45.6 Gy/0
PAN	–	–	–	–	–	36 Gy
Central shielding	Yes	–	Yes	No	Yes	No
ICBT (Point A dose)	Tandem & ovoids (24 Gy/4 fractions)	–	Vaginal cylinder (15.3 Gy/3 fractions)	–	Vaginal cylinder (30 Gy/6 fractions)	–
Salvage radiotherapy for recurrence	–	–	ISBT (48.3 Gy /7 fractions, HR-CTV D90)	–	ICBT with vaginal cylinder (20 Gy/5 fractions, 5 mm depth)	–
Cumulative EQD2 (primary + salvage)^b^						
Rectum (D2cm^3^)	96.5 Gy	50 Gy^c^	136.8 Gy	50 Gy^c^	121.2 Gy	45.6 Gy^c^
Bladder (D2cm^3^)	64.3 Gy	50 Gy^c^	135.4 Gy	50 Gy^c^	71.6 Gy	45.6 Gy^c^
Estimated dose to the fistula lesions	96.5 Gy (rectum)	50 Gy^c^** (rectum)**	135.4 Gy (bladder)	50 Gy^c^(bladder)	121.2 Gy (rectum), 71.6 Gy (bladder)	45.6 Gy^c^ (ureter)
Site of fistula^d^	Vagina and rectum	Vagina and rectum	Vagina and ureter	Vagina and bladder	Vagina and bladder / vagina and rectum	Right ureter and right common iliac artery

*CCRT* concurrent chemotherapy and radiotherapy, *EQD2* equivalent dose in 2 Gy fractions, *HR-CTV* high-risk clinical target volume, *ICBT* intracavitary branchytherapy, *ISBT* interstitial brachytherapy, *PAN* para-aortic nodes, *RT* radiotherapy, WP/CS-*WP* whole pelvis/central shield-whole pelvis

^a^Patient 2 column indicates the patient whose data were not further investigated in the retrospective case series study and data from PMS study are listed

^b^Doses through CS-WP external beam radiotherapy were not included in cumulative dose calculations

^c^Estimated as prescribed doses of external beam radiotherapy

^d^Fistula could cover multiple sites

**Table 5:** Characteristics of fistulas in patients with cervical cancer who developed a pelvic fistula with bevacizumab

	Patient 1	Patient 2^a^		Patient 3	Patient 4	Patient 5^c^		Patient 6
Site of fistula^b^	Vagina and rectum	Vagina and rectum		Vagina and ureter	Vagina and bladder	Vagina and bladder	Vagina and rectum	Right ureter and right common iliac artery
Worst grade of fistula	3	3		3	2	2	3	4
Time from start of bevacizumab to onset of fistula symptoms, days	97	72		158	172	21	77	131
Diagnostic method	CT	–		MRI	MRI	MRI	MRI	CT
Blood test results just prior to first bevacizumab administration								
Hemoglobin, g/dL	11.2	NA		9.6	9.7	11.8		13.6
Platelets, × 10^4^/mm^3^	42	NA		27.4	17.5	34.7		26.9
Albumin, g/dL	3.4	NA		3.3	3.4	3.1		4.3
Creatinine, mg/dL	0.57	NA		0.64	0.98	0.73		1.30
C-reactive protein, mg/dL	0.97	NA		0.34	1.97	3.02		0.52
Blood test results just prior to fistula onset								
Hemoglobin, g/dL	10.4	NA		9.3	9.5	8.3	10.5	10.7
Platelets, × 10^4^/mm^3^	30.3	NA		20.9	22	25.8	33.3	45.5
Albumin, g/dL	2.6	NA		3.3	4	–	3.2	3.3
Creatinine, mg/dL	0.4	NA		0.58	1.02	0.04	0.72	1.13
C-reactive protein, mg/dL	10.75	NA		1.16	1.91	4.79	2.38	–
Bevacizumab discontinued	Yes	Yes		Yes	Yes	No	Yes	Yes
Invasive intervention for fistula management	Colostomy	Hartmann's operation		Ileal conduit construction	None	None	Colostomy	Right external iliac artery stent graft insertion, right internal iliac artery embolization, bilateral percutaneous nephrostomy
Fistula outcome	Remission	Remission		Recovered	Not recovered	Not recovered	Not recovered	Recovered
Days from fistula onset to outcome	695	3		671	69	348	293	1212
Treatment of cervical cancer after fistula diagnosis	Yes (paclitaxel + cisplatin)	NA		BSC	BSC	BSC		BSC
Survival status at the time of data collection^d^	CC death	N/A		Alive	CC death	CC death		Alive

*BSC* best supportive care, *CC* cervical cancer, *CT* computed tomography, *FGF* female genital fistula, *MRI* magnetic resonance imaging, *NA* not available

^a^Patient 2 column indicates the patient whose data were not further investigated in the retrospective case series study and data from PMS study are listed

^b^Fistula could cover multiple sites

^c^Patient 5 had two events

^d^All deaths were caused by the underlying cervical cancer

The time from first administration of bevacizumab until fistula development ranged from 21 to 172 days (median 97 days). Treatment with bevacizumab was discontinued in all patients with fistula (Table 5).

All six patients with pelvic fistulas, including the one without additional investigation, had squamous cell carcinoma, recurrent disease, tumor lesions at baseline, and a history of radiotherapy (Tables 3 and 4). All six patients also had a history of radiotherapy and five patients (four of the investigated five) had undergone surgery (hysterectomy in four of the five). One patient was a smoker, two were non-smokers, while smoking history was unknown in the others.

Five of the six patients underwent CCRT prior to bevacizumab-containing chemotherapy, and two of these patients underwent re-irradiation at the time of recurrence before bevacizumab. Three patients (1, 3, and 5) were treated with definitive CCRT as a primary treatment, and two of these patients (3 and 5) received secondary radiotherapy with brachytherapy as a salvage treatment for local recurrence. One of these two (patient 5) had also undergone simple hysterectomy as secondary salvage treatment after re-irradiation. For these three patients (1, 3 and 5), cumulative radiation doses to the organs that subsequently developed fistula were high compared with dose limits stated in guidelines [15, 16].

Three patients (patients 2, 4 and 6), including the one without additional investigation, were treated with radical hysterectomy followed by postoperative radiotherapy/CCRT as an initial treatment. Patients 2 and 4 also received whole pelvic external beam radiotherapy (EBRT) of 50 Gy. Patient 6 received both whole pelvic and para-aortic nodal irradiation with two separate radiotherapy plans (Table 4). No dose overlapping occurred, confirmed by referring to actual treatment plan data.

Both patients who received 50 Gy of EBRT without central shielding (patients 2 and 4) received radiotherapy after radical hysterectomy, followed by bevacizumab combination chemotherapy for pelvic recurrence. Patient 6 had a history of bilateral ureteral stent placement after radical hysterectomy. After four cycles of treatment with paclitaxel + carboplatin chemotherapy and bevacizumab, this patient developed a right uretero-common iliac artery fistula, which was successfully treated with right external iliac artery stent graft insertion, right internal iliac artery embolization, and bilateral percutaneous nephrostomy. This patient had a complete response to chemotherapy + bevacizumab, and was alive and recurrence-free at 1212 days after the onset of fistula formation.

Levels of hemoglobin, creatinine, albumin, and platelets were generally similar at the onset of fistula (Table 5), although albumin levels were low (< 3.5 g/dL) prior to fistula formation in four of the five patients. Two patients (patients 1 and 3) had increased C-reactive protein levels between the start of bevacizumab and fistula diagnosis (from 0.97 to 10.75 mg/dL in one and from 0.34 to 1.16 mg/dL in the other).

## Discussion

This real-world, PMS study in Japanese patients with advanced/recurrent cervical cancer receiving bevacizumab and chemotherapy showed an overall rate of all-grade fistula of 4.2% (95% CI 1.56–8.96) and a rate of grade ≥ 3 fistulas of 3.5%. This contrasts with rates of 15% for all-grade fistula and 6% for grade ≥ 3 fistula observed in the GOG 240 study [12].

Although fistulas in gynecological malignancy are rare, they are known complications of this type of cancer, and can lead to substantial physical and psychological morbidity [17–19]. Vesicovaginal and enterovaginal fistulas are common, but ureterovaginal, enterovesical, rectovaginal, and enterocutaneous fistulas also occur [17, 19]. Symptoms include urinary leakage or fecal discharge through the vagina, bleeding, fever and pain [17–19], and fistulas can be diagnosed and examined using various imaging methods [19]. Fistulas can be managed symptomatically or surgically [19].

Inhibition of angiogenesis by bevacizumab may be involved in the development of fistulas [20], and several studies of cervical cancer patients have reported fistula development following bevacizumab treatment [21, 22]. Aside from bevacizumab, other risk factors for fistulas include radiotherapy (rectal dose ≥ 76 Gy), previous surgery, tumor stage (III or IV vs. I or II), ureteral stents, nutritional status and inflammatory bowel disease [19, 22–26]. In the present study, all patients who developed a fistula had undergone previous radiotherapy, which was also the case for all the patients who developed grade 3 fistula in the final analysis of the GOG 240 study [12]. In the primary results of the phase II CECILIA study, 17 of 150 patients had perforation/fistula (11.3%) and 16 of the 17 patients with fistula (94.1%) had received previous radiotherapy [27].

Radiotherapy results in an increase in fibrosis, endothelial abnormalities and endothelial apoptosis, and can result in the development of complex wounds, the healing of which can be inhibited by concomitant administration of an angiogenesis inhibitor such as bevacizumab [28]. Standard radiotherapy procedures used in Japan differ from those in Western countries in several ways. Use of central shielding for the later part of EBRT is the most remarkable distinction [29, 30]. In addition, the prescribed brachytherapy doses in Japan are lower than those recommended in international guidelines [15, 16, 29, 30]. Consequently, definitive radiotherapy could be less intensive, and this may help to explain the (low) incidence of fistula formation in this Japanese real-world series compared with GOG 240 study, although direct inter-trial comparison is not appropriate. It is also worth noting that patient backgrounds differed between the current study and GOG 240 [12], with a lower proportion of patients in our study undergoing previous pelvic radiotherapy (64.1% vs. 80%) and a high proportion having a Gynecologic Oncology Group performance status of 0 (73.2% vs. 58%). It is possible that post-marketing risk management activities based on the results of GOG 240 may have influenced patient selection for bevacizumab in the current PMS study.

While the vagina has been shown to have a low level of complications after definitive radiotherapy [31, 32], vaginal stenosis and vesicovaginal/rectovaginal fistulas are known complications following radiotherapy [33, 34]. Fistula is regarded as a relatively rare complication after radiotherapy. In a study of 62 patients with stage IIB or IIIB cervical cancer treated with radiotherapy, fistulas developed in two patients (3.2%) [35], while another study of patients with stage IVA disease treated with radiotherapy reported a fistula rate of 1/26 (3.8%) [36]. Radiotherapy dose to the rectal wall is regarded as the most important predictive factor for developing fistulas. In the large international prospective observational EMBRACE study, the risk of rectal fistula was 12.5% at 3 years in patients who received > 75 Gy, but only 0–2.7% in those with lower dose of the rectum (D2cm^3^) [37]. In the present case series, two of three patients who developed rectovaginal fistula received high cumulative doses of radiation (96.5 Gy, 121.2 Gy) to the rectum. The high rectal doses might have affected the onset of the fistulas in addition to bevacizumab administration. On the other hand, it should be kept in mind that unpredictable fistula formation can occur even when the cumulative vesicorectal radiation dose does not exceed the tolerable threshold, as occurred in cases 2, 4, 5, and 6 in the current series [38].

Injury during surgery for cervical cancer has also been implicated in the development of fistulas; in a retrospective review of 323 surgical procedures for cervical cancer, fistulas developed in 2.7% of patients [18]. In our study, five of the six patients with fistulas had undergone pelvic surgery, including surgery at a total radiation dose of approximately 50 Gy, although we were not able to compare the surgical history of patients with and without fistula formation. The patient in our study who developed a right uretero-common iliac artery fistula had a history of ureteral stenting. Previous research has shown a relationship between invasive procedures, such as ureteral stents and local biopsy, and fistula formation [22].

Regarding the risk of fistula in patients with poor nutritional status, in one study of patients with recurrent and metastatic cervical cancer, lower albumin levels were an independent predictor of fistula formation [23]. Similarly, in our study, four of the five patients with evaluable data in the case series study had albumin levels of < 3.5 g/dL at the time of fistula diagnosis. Because patients with advanced recurrent disease are generally undernourished, it may be advisable to measure albumin levels before administering bevacizumab. However, we did not investigate albumin levels in patients without fistulas, so clear conclusions cannot be drawn about the relationship between albumin levels and fistula development in our study. Another study stratifying patients with locally advanced cervical cancer by baseline body mass index (BMI) found that those with a BMI < 18.5 kg/m^2^ had an increased risk of grade 3 or 4 fistulas compared with those with a BMI > 24.9 kg/m^2^ (11.1% vs. 8.8%; *p* = 0.05) [24]. In the present study, 17.6% of the overall patient cohort had a BMI < 18.5 kg/m^2^, and two of the six patients who developed a fistula had a BMI < 18.5 kg/m^2^.

There are limited published data on the management of fistulas in patients with cervical cancer. However, the American Society of Clinical Oncology clinical practice guidelines recommend surgical repair, with a diverting colostomy the treatment of choice for rectovaginal fistulas [39]. For ureterovaginal fistulas, these guidelines recommend creation of a ureterointestinal conduit or decompression of the ureters using bilateral percutaneous nephrostomies [39].

This study has several limitations. Risk factors for fistulas cannot be determined from our study which was a PMS study without a predetermined statistical sample size. While we were able to examine more closely the clinical and treatment characteristics of five patients with fistulas who had received prior treatment, including with radiotherapy, no comparable data were available for patients without fistulas, and therefore risk factors cannot be identified by comparison. There was no control group in this study, so the impact of bevacizumab per se on fistula development could not be confirmed.

In conclusion, this Japanese PMS study found a lower rate of fistula development in patients receiving bevacizumab plus chemotherapy than that seen in studies conducted in the US and Europe, with the upper limit of the 95% CI for the incidence of pelvic fistulas in this study not exceeding the incidence reported in the GOG 240 study [12]. The results of the PMS study did not reveal any new safety concerns not seen in clinical trials, but given the potential for fistula, bevacizumab should be administered after a comprehensive evaluation of the bladder/rectal dose of radiotherapy as pretreatment, of invasive treatment, such as surgical therapy, and of nutritional status. In addition, the possibility of unpredictable fistula formation despite such a comprehensive evaluation should be shared with the patient for benefit-risk assessment and treatment decision-making. Further research is needed to identify risk factors for fistula development in patients receiving bevacizumab for cervical cancer.

## Supplementary Information

Below is the link to the electronic supplementary material.

Supplementary file1 (PDF 400 KB)
