# Supplementary material for: Incidence of fistula occurrence in patients with cervical cancer treated with bevacizumab: data from real-world clinical practice
Source: Int J Clin Oncol. 2022 Jun 27;27(9):1517–28. doi: 10.1007/s10147-022-02196-8 (PMC9393147; doi:10.1007/s10147-022-02196-8)
Supplement: Supplementary file 1 — Supplementary file1 (PDF 400 KB) [file 10147_2022_2196_MOESM1_ESM.pdf]

Incidence of Fistula Occurrence in Patients with Cervical Cancer Treated with Bevacizumab: Data from Real-world Clinical Practice

International Journal of Clinical Oncology

Toru Sugiyama, MD PhD[1], Noriyuki Katsumata, MD PhD[2], Takafumi Toita, MD PhD[3], Masako Ura, BS[4], Ayaka Shimizu, BPharm[5], Shuichi Kamijima, MD PhD[6], Daisuke Aoki, MD PhD[7]

Correspondence to: Toru Sugiyama, MD PhD

St. Mary's Hospital, 422 Tsubukuhonmachi,

Kurume, Fukuoka, 830-8543, Japan

Email: sugiyamatoru0802@yahoo.co.jp

Online Resource Table S1. Information collected from patient records in the case series study

Major item	Sub-item
**Patient background**	
Smoking history	Presence or absence/details
Past history and coexisting diseases	Presence or absence/details
**Previous surgery before bevacizumab**	
History of surgery	Presence or absence
Surgical procedure	Name
	Operation time
	Intraoperative blood loss (mL)
**Radiation therapy/external-beam radiation prior to bevacizumab (at initial treatment)[a]**	
External irradiation	Presence or absence
Central shielding	Presence or absence
	If yes, when
Radiation therapy (irrespective of central shielding)	Irradiation start date to irradiation end date
	Irradiation method
	Irradiation field
	X-ray energy
	Number of fractions
	Dose per fraction (Gy)
	Total dose (Gy)
**Radiation therapy/intracavitary irradiation before bevacizumab (at initial treatment)**	
Intracavitary irradiation[a]	Presence or absence
	Irradiation start date to irradiation end date
	Dose per fraction (Gy): Point A dose/ HR-CTV D90
	Number of fractions
	Total dose (Gy):
	Total rectal dose (Gy): ICRU38/ D2cm^3
	Total bladder dose (Gy): ICRU38/ D2cm^3
	Types of Ovoid Applicators
	HYBRID brachytherapy: Yes/No
	If yes, no. of applicators
**Radiation therapy/interstitial irradiation before administration of bevacizumab (at initial treatment)**	
Interstitial irradiation[a]	Presence or absence
	Irradiation start date to irradiation end date

	Dose per fraction (Gy)
	Number of fractions
	Total dose (Gy)
	Total rectal dose (Gy)
	Total bladder dose (Gy)
**Radiation therapy for recurrent tumor before bevacizumab**	
Radiation therapy[a]	Presence or absence/details
For external, intracavitary or interstitial irradiation	Irradiation start date to irradiation end date
	Overlap with the extent of initial radiation therapy
	Irradiation method (only in case of external irradiation)
	Irradiation field (only in case of external irradiation)
	X-ray energy (only in case of external irradiation)
	Dose per fraction (Gy)
	Number of fractions
	Total dose (Gy)
For intracavitary or interstitial irradiation	Total rectal dose (Gy)
	Total bladder dose (Gy)
For intracavitary irradiation only	Types of ovoid applicators
	HYBRID (intra-tissue irradiation applicator added): Yes/no
	If yes, number of units and site
**Presence or absence of concurrent chemoradiotherapy (CCRT) prior to bevacizumab**	
Concurrent chemoradiotherapy	Presence or absence
	Concomitant drug
	Duration of administration
**Presence or absence of recurrent tumor at the start of bevacizumab**	
Recurrent tumor	Presence or absence
	Site of recurrence
	Recurrence date
Recurrent tumor	Presence or absence of recurrent tumor biopsy
**Immediately before bevacizumab administration**	
Before the first dose of bevacizumab or just before the onset of fistula	Date of inspection
	Hemoglobin (g/dL)
	Platelet ($\times 10^4/mm^3$)
	Albumin (g/dL)

	Creatinine (mg/dL)
	HbA1c (%)
	CRP (mg/dL)
	Urine protein (dipstick method)
**Information about bevacizumab combination chemotherapy**	
Number of bevacizumab-treated cycles	Number of bevacizumab-treated cycles
Tumor shrinkage effect	Response
**Information about the fistula**	
Diagnostic information	Diagnostic method
	Date of diagnosis
Additional measures for fistulas	Presence or absence
	Details and date of treatment
Current outcome of fistula	Date of outcome
	Outcome
**Patient outcome information**	
Treatment of cervical cancer after fistula	Presence or absence
	Treatment content
	Duration of treatment
Survival	Outcome
	Confirmation date (last survival confirmation date or death date)

[a]The digitally reconstructed radiographs and dose distribution map for each radiation treatment was requested

CRP, C-reactive protein; HbA1c, glycated hemoglobin; HRCTV, high-risk clinical target volume; ICRU, International Commission on Radiation Units & Measurements.
